# Endocrine Disruptors and Breast Cancer: A Comprehensive Review

**DOI:** 10.3390/biomedicines13112774

**Published:** 2025-11-13

**Authors:** Luiza Czaczkowska, Ewa Jabłońska, Wioletta Ratajczak-Wrona

**Affiliations:** Department of Immunology, Medical University of Białystok, 15-269 Białystok, Poland; immu-no@umb.edu.pl

**Keywords:** breast cancer, EDC, Xenoestrogens, hormone receptors, ECHA

## Abstract

Breast cancer is one of the most prevalent malignancies affecting women worldwide. Among environmental risk factors, increasing attention has been given to endocrine-disrupting chemicals (EDCs), which can interfere with hormonal signaling pathways. Chronic exposure to these compounds, even at low doses, may lead to molecular changes that initiate carcinogenesis or promote tumor progression. Owing to EDCs’ resistance to degradation and ability to bioaccumulate in organisms and the environment, they pose a growing concern for human health. They can mimic or block natural hormones by binding to receptors, such as estrogen, progesterone, aryl hydrocarbon, or thyroid-stimulating receptors, disrupting hormone synthesis, secretion, and metabolism. They have shown the ability to initiate carcinogenic changes in breast tissue or accelerate cancer progression. This review focuses on the relationship between EDC exposure and breast cancer, examining both their mechanisms of action and long-term health effects. Compounds such as polychlorinated biphenyls, parabens, phenols, 2,3,7,8-tetrachlorodibenzo-p-dioxin, diethylhexyl phthalate, and bisphenol A, which are frequently encountered in everyday products, are discussed in detail. By presenting European Union guidelines and exploring EDCs’ biological activity and pathways of endocrine disruption, we aimed to raise awareness of their potential risks and emphasize the need for further research.

## 1. Introduction

### 1.1. Endocrine-Disrupting Chemicals

Endocrine-disrupting chemicals (EDCs) are a heterogeneous group of many substances that affect the ability and function of healthy cells and hormones. This group includes organic and chemical substances, as well as xenobiotics and pesticides. They can disturb hormone-induced processes and interfere with endocrine organs and organs related to endocrine pathways. They bioaccumulate in living organisms and the environment and are deposited in soil and water. EDCs have a lipophilic character, which helps them, among other things, penetrate membranes and accumulate in adipose tissue. Their potential ability to mimic 17-β-estradiol, influenced by integrins, which are adhesion molecules, and the cytoskeletal network, suggests that adherent cells may be targets of these substances. Furthermore, different substances can interact with the same genes and contribute to the molecular processes of carcinogenesis [1,2,3,4,5].

Among this group of compounds are xenoestrogens. These compounds can exert a wide range of estrogenic effects. They may act as either agonists or antagonists toward the nuclear estrogen receptor, especially estrogen receptors α and β. Usually, substances show a response curve that reflects exposure to the compound—a straight line that increases proportionally with the dose. However, they can also produce biphasic or nonmonotonic dose–response (NMDR) curves. This means that the relationship between dose and effect is not necessarily linear, and each compound in this group may be metabolized differently. Some may even cause effects that are opposite to what is expected. Adverse effects may not occur at the highest concentration but, for example, after administration of 50% of the acceptable daily intake. Therefore, the minimum effective concentrations of these compounds should be approached with caution [6].

Experts in the field of endocrinology and research on endocrine-disrupting chemicals have detailed 10 key characteristics of EDCs (KCs). They state that EDCs can (KC1) interact with or activate hormone receptors, (KC2) antagonize hormone receptors, (KC3) alter hormone receptor expression, (KC4) alter signal transduction in hormone-responsive cells, (KC5) induce epigenetic modifications in hormone-producing or hormone-responsive cells, (KC6) alter hormone synthesis, (KC7) alter hormone transport across cell membranes, (KC8) alter the hormone distribution or circulating levels of hormones, (KC9) alter hormone metabolism or clearance, and (KC10) alter the fate of hormone-producing or hormone-responsive cells [1].

Some EDCs are also known to be obesogens. They increase fat accumulation by promoting adipogenesis. Many factors contribute to obesity. One of them may be obesogens, environmental chemicals that disrupt hormonal regulation and metabolic processes. It is known that obesity is closely linked to various diseases. It is also a risk factor for breast cancer [7,8].

**Figure 1:**
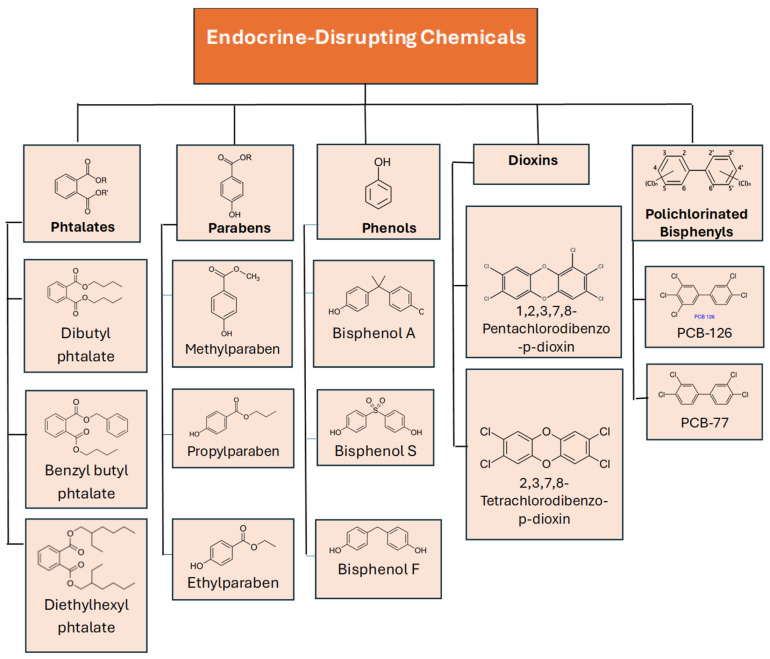
Group of endocrine-disrupting chemicals, with groups of compounds written in bold font.

Endocrine-active compounds include many groups of substances. They include phytoestrogens, mycotoxins, parabens, dioxins, pesticides, organochlorine pesticides, natural hormones, phthalates, polychlorinated compounds, alcohols, and bisphenols. The compounds discussed in this article belong to the groups included in Figure 1. These substances are commonly used in various industries as rubber chemicals, plasticizers, industrial solvents, and lubricants. They are present in PVC and materials that are used in buildings and construction sites, such as sinks, mirrors, walls, paints, and glues. They are also found in personal care products, acne treatment products, air fresheners, cleaning products, and children’s toys and crayons [1,9,10].

The European Chemicals Agency (ECHA) has been keeping an endocrine disruptor (ED) assessment list, which was first created in February 2013 and was last updated on 21 January 2025. The list contains 127 substances. There were no changes since the update on 21 October 2024. The outcomes of the investigations were labeled as ED ENV (Environment); ED HH (regarding human health), Under Development BPR (Biocidal Product Regulation), SEV (Severe), or other; inconclusive; postponed; and not ED. The authority carrying out the surveillance of endocrine disturbances can be either the ECHA, the European Commission, or a European Union (EU) member state’s competent authority [11].

In 2018, the European Commission introduced Regulation (EU) 2018/605, which established criteria for identifying endocrine-active substances in accordance with the provisions of Regulation (EC) No 1107/2009. According to this regulation, a substance can be considered endocrine-active if it possesses certain characteristics: It has an adverse effect on the body, leading to impaired physiological functions, reduced adaptive capacity, or increased susceptibility to other environmental factors. It affects the endocrine system by altering its function. To be classified as an EDC, there must also be a scientifically documented causal relationship between the effect on the endocrine system and the observed adverse effects [12].

Commission Regulation (EU) 2024/2711 amends Annexes II and V to Regulation (EC) No. 396/2005 by setting new maximum residue limits (MRLs) for thiacloprid in certain food products. Thiacloprid, an insecticide, has raised safety concerns regarding its use. Therefore, pesticide residues and maximum residue levels have been reduced for many products to 0.01 mg/kg. This decision is based on risk assessments carried out by the EFSA, which indicate the potential risks related to thiacloprid. The regulation will apply from 12 May 2025 in all EU member states [13].

Some EDCs have been labeled as carcinogens, and some have been tested for this. These substances can function as carcinogens and cause damage to the genome. They also participate in the growth and progression of cancer and affect the synthesis, secretion, and metabolism of hormones [14].

In the EU, there are several regulations governing the use of potentially carcinogenic substances. The International Agency for Research on Cancer (IARC) classifies xenobiotics based on their potential carcinogenicity. In terms of evidence, it divides substances into five groups, with Group 1 being a group of substances with proven carcinogenic effects on humans. REACH (EC 1907/2006) requires the registration, evaluation, and authorization of chemical substances, including xenobiotics. The CLP Regulation (EC 1272/2008) harmonizes the classification and labeling of carcinogenic substances. Directive 2004/37/EC regulates the protection of workers from the effects of carcinogens and mutagens [15,16,17].

In 2012, the UNEP and WHO, in collaboration with international experts, produced the State of the Science of Endocrine Disrupting Chemicals, which lists basic concerns on human and wildlife health in relation to those substances. In this State of Science, many long-term effects have been included. Some of them raise concerns about disturbing reproductive ability and some about other possible diseases, including breast cancer [18].

### 1.2. Breast Cancer

**Figure 2:**
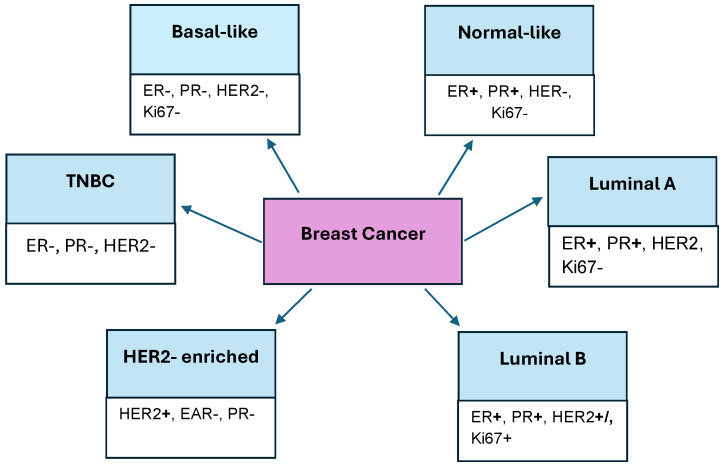
Types of breast cancer and their ligands.

Breast cancer is the most common type of cancer among women. At the 12th International Breast Cancer Conference in 2020, cancer was divided into four molecular subtypes, which are determinants of treatment choice. They were divided based on their expression of hormone receptors or lack of them, which is presented in Figure 2. Estrogen receptor (ER), progesterone receptor (PR), Human Epidermal Growth Factor Receptor 2 (HER2), and overexpression of the nuclear protein Ki67 were taken into account. The subtypes of breast cancer are Luminal A, Luminal B, HER2-enriched, and Triple-Negative breast cancer (TNBC). The basal-like subtype lacks the expression of ER, PR, HER2, and Ki67 receptors. The normal-like subtype of breast cancer contains ERs and PRs but lacks HER2 and Ki67. HER2 shows expression of the HER2 receptor, but lacks it in ERs and PRs. The luminal subtypes express ERs and PRs, while the Luminal A subtype can also express the Ki67 receptor and HER2, in contrast to Luminal B, which does not express it [19,20,21,22].

In 2019, the WHO established a classification of breast tumors. The two main groups are breast carcinomas and neuroendocrine neoplasms (NENs). The first is characterized by molecular subtypes, as mentioned above. Furthermore, newly added data support the differences between ER and HER2, and their pathogenesis [23].

**Figure 3:**
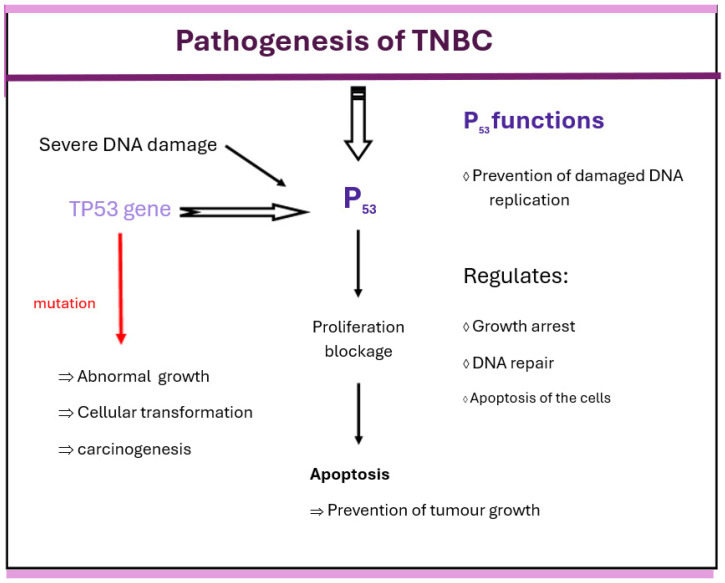
Pathogenesis of TNBC in relation to P_53_.

P53, which is regulated by TP53, plays an important role in the pathogenesis of TNBC. Its pathogenesis is presented in Figure 3. The P53 protein regulates cell growth arrest, DNA repair, and apoptosis. Temporary arrest of cell cycle progression, correction of damaged DNA, and prevention of DNA replication are important functions of this protein. P53 also promotes DNA repair, and once this is completed, the cell cycle resumes. Severe DNA damage that cannot be repaired results in apoptosis. In cells with wild-type p53, DNA damage can trigger the p53 protein to halt cell proliferation and promote apoptosis, thus helping to prevent the development of tumors. When a mutation occurs, TP53 loses its protective role in cell regulation, leading to abnormal cell growth, cellular transformation, and carcinogenesis. Most TNBC patients with TNBC have TP53 mutations [3,24].

There is growing evidence that obesogens may influence the risk of developing breast cancer. The few publications on the increased risk of TNBC in premenopausal women who are also obese suggest a role for obesogens in this process. Adipogenesis correlates with the poorest prognosis in TNBC [8].

This review aimed to determine the carcinogenic and possible long-term effects of these compounds in relation to breast cancer. In this review, we will take a closer look at xenoestrogens in correlation with breast cancer and review a few of the many possible long-term effects on human health. We will also review the mechanisms discussed above, which are enhanced by endocrine-active compounds. The examination of the mechanisms by which these compounds influence the risk of breast cancer will be studied. Phenols, parabens, polychlorinated biphenyls, diethylhexyl phthalate, and 2,3,7,8-tetrachlorodibenzo-p-dioxin will be discussed.

## 2. Materials and Methods

There was a total of 53,178 articles related to the phrase “Breast cancer,” 78 related to “EDC induced breast cancer,” and 4090 results related to “EDC” when we searched on the PubMed and Google Scholar platforms. Inclusion criteria for these articles included references to any further-mentioned substances—polychlorinated biphenyls, parabens, phenols, 2,3,7,8-tetrachlorodibenzo-p-dioxin, diethylhexyl phthalate, and bisphenol A—as well as a relation to breast cancer and long-term effects. Meta-analyses, cohort studies, epidemiological studies, integrated bioinformatic analysis, research articles, in vitro studies, and case–control studies with relevance for breast cancer and EDCs were of interest. Articles published between 1995 and 2025 were considered, with the majority having been written in the last five years. Excluded articles did not contain enough content or research data to support their claims. Excluded articles also did not add new specific information. The legal acts and regulations in this article were researched on a website dedicated to European Union legal regulations. The EUR-Lex website was used for this purpose. The compounds mentioned above were the focus of searches for regulations concerning their maximum allowable concentrations, depending on their use and potential contact with the human body.

## 3. Discussion

The relationship between EDCs and breast cancer pathogenesis depends on estrogenic overactivity. Xenoestrogens are known to have estrogen-mimicking functions, causing hormonal disruption by binding to estrogen receptors. There are three estrogen receptors: ERα, ERβ, and G-protein-coupled estrogen receptor (GPER) [3,25,26,27,28].

**Figure 4:**
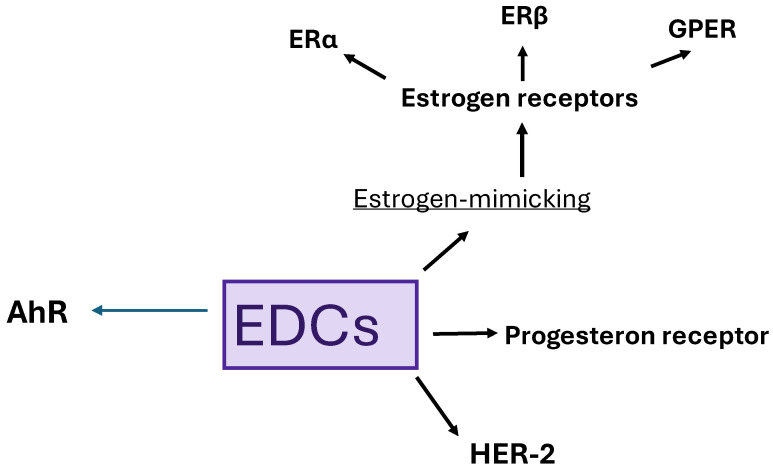
Receptors of EDCs.

It is also impossible not to mention the aryl hydrocarbon receptor (AhR), a transcription factor. The AhR receptor is an antagonist, and its ligand, which activates it, causes agonist action. This receptor plays an important role in the development of immune responses and signaling in endogenous metabolism, as it is widely expressed in many organs. AhR activity is dependent on signal transducers such as c-Src and NF-κB. Xenoestrogen ligands for AhR that mimic estrogens affect signaling via different pathways. They bind to their receptors and stimulate both genomic and non-genomic activation. All receptors related to EDCs are presented in Figure 4, with the main groups being estrogen, progesterone, HER-2, and AhR receptors [29,30].

Several studies present evidence on breast cancer subtypes and EDC exposure. One study used Mendelian randomization (MR) to examine the effect of bisphenols, parabens, and phthalates on the overall breast cancer risk. Using this method, the study found a positive association between n-butyl paraben and Luminal A breast cancer. A positive association was also found between mono-iso-butyl phthalate, a metabolite of diisobutyl phthalate, and TNBC. However, no positive associations were found between monomethyl phthalate and Luminal B breast cancer, and no association between phthalates and breast cancer risk was suggested. The role of BPA in the pathogenesis of breast cancer is widely debated. Articles both supporting and refuting this risk are readily available [31,32].

One study focused on the risk of Luminal A breast cancer in black women, in correlation with parabens. They used cancer cell lines for Luminal A (MCF-7 and HCC1500), as well as for Luminal B (BT-474 and MDA-MB-175-VII). The results of their study show an interesting relation. In general, the HCC1500 (West African ancestry) Luminal A breast cancer cell line seems to be more sensitive to parabens than the MCF-7 (European ancestry) Luminal A breast cancer cell line (Tapia JL et al. 2023 [33]). However, the number of studies examining the exact mechanism between paraben correlation (as well as other EDCs) and breast cancer is limited. Research on subtypes of breast cancer is an underexplored area that should generate more interest [33].

### 3.1. Poyichlorinated Biphenyls (PCBs)

PCBs were commonly used as hydraulic lubricants and heat transfer fluids, as well as in dust-reducing agents and as antioxidants in paint and in carbonless copy papers. Currently, they can be released into the environment as a result of waste degradation and are still present in the materials of old buildings. New PCB production is banned globally, but legacy use still exists in old transformers, heat transfer fluids, and industrial equipment. Appropriate legal regulations define the standards for the occurrence of these compounds, both in the environment and in everyday products, such as food. Regulation 2023/915 specifies the levels of PCB contamination in various food groups, including meat, fish, dairy products, eggs, and feed. For meat, it is 0.09 ng/g, for fish, 0.75 ng/g, while for dairy products, it is 0.01 ng/g [34,35].

They have unique characteristics that contribute to their endocrine-disrupting roles. They are hydrophobic and resistant to degradation; therefore, they exhibit high bioaccumulation and biomagnification potential in the environment. PCBs are agonists of estrogen receptors and aryl hydrocarbon receptors but act as antagonists of androgen receptors. Their association with AhR causes the inhibition of ERs. The current scientific literature does not provide definitive evidence that polychlorinated biphenyls (PCBs) act as direct agonists or antagonists of the progesterone receptor (PR). Pathomechanism is associated with the p53 protein, Ki-67, and through their relation to AhR and Cytochrome P450, PCBs are directly related to oxidative stress. The negative prognosis of breast cancer may be caused by excessive production of reactive oxygen species (ROS), disorders of intercellular communication, and inhibition of apoptosis in cancer cells. The overexpression of HER-2 is associated with lymph node metastasis in breast cancer [34,36,37,38,39,40].

PCBs are recognized as endocrine-disrupting chemicals (EDCs) that are capable of interfering with hormonal signaling pathways; however, their specific interaction with PRs remains unclear. Some studies have suggested that PCBs indirectly influence PR expression or function. For instance, PCBs have been shown to modulate the expression of hormone receptors in certain tissues, potentially affecting PR activity. However, these effects are not necessarily due to direct binding to the PR but may result from broader disruptions in endocrine signaling [39,41].

PCBs are divided into two groups: dioxin-like (DL-PCB) and non-dioxin-like (NDL-PCB) PCBs. DL-PCBs can activate the AhR. Compounds from both groups also have the ability to activate transcription via human thyroid hormone receptor β and estrogen receptors. Some of these substances can also promote breast cancer metastasis to the lymph nodes. Furthermore, a higher percentage of polychlorinated biphenyls in adipose tissue has been linked to cancer recurrences [39,42,43].

**Figure 5:**
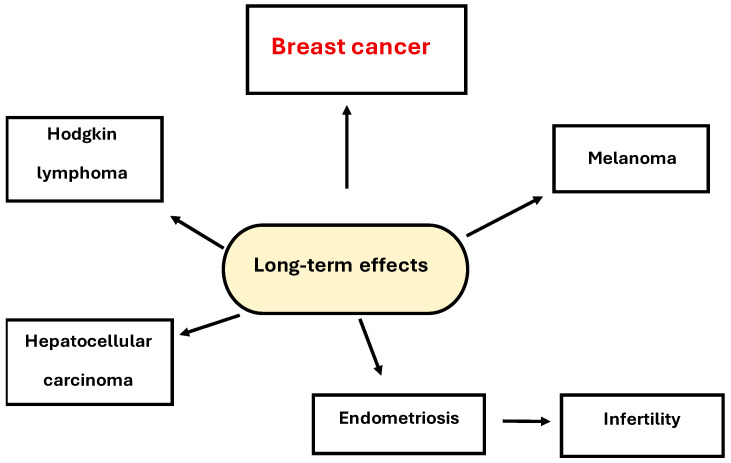
Long-term effects of PCBs.

The long-term effects of PCBs are shown in Figure 5. One of the long-term effects of these compounds, in addition to breast cancer, is infertility caused by endometriosis. There are also many reports indicating their role in the development of non-Hodgkin lymphoma, melanoma, and hepatocellular carcinoma [42,43].

### 3.2. Parabens

Parabens are used as food preservatives in fruits, cooking oils, fast foods, and personal care products, such as cosmetics, shampoos, toothpastes, and pharmaceutical products. They can bioaccumulate and are present in the adipose and breast tissues of women. European Union regulations have introduced maximum permissible concentrations of parabens in cosmetics and food. No. 1223/2009 specifies concentrations of methylparaben and ethylparaben at a maximum of 0.4% individually, 0.8% in mixtures, and propylparaben and butylparaben at a maximum of 0.14% in total. Additionally, a ban on the use of these compounds in cosmetics intended for children under 3 years of age has been introduced. Separate regulations have also been introduced regarding the use of polychlorinated biphenyls in food. Regulation (EC) No 1333/2008 pertains to food additives. According to this regulation, propylparaben (E216) and its sodium salt (E217) were banned from food in 2006. Isopropylparaben, isobutylparaben, phenylparaben, benzylparaben, and pentylparaben were banned as a result of Commission Regulation (EU) No. 358/2014. In addition to their carcinogenic properties, they also have other side effects on human health, which should raise an alarm on the safety of their use in cosmetics [33,44,45,46,47,48].

Currently, according to the WHO, they are not classified as carcinogenic. There are a few types of them that are tagged as hazardous: butylparaben (BP), propylparaben (PP), and methylparaben (MP). These compounds are especially dangerous during pregnancy, because they disrupt thyroid and reproductive hormones and cause chronic inflammation caused by oxidative stress [33,44,49,50].

Like many xenoestrogens, they have the ability to bind to estrogen receptors. Estrogens, owing to their similar structure to these compounds and the competitive binding of parabens, are displaced from their Erα and Erβ receptors. However, they might have different effects on organisms than 17β-estradiol. They can also block the progesterone, androgen, and glucocorticoid nuclear receptors. This means that parabens can act as antagonists to AhR, AR, and PRG receptors, and as weak agonists of ERα and ERβ [44,48,50].

In addition to their estrogen-related mechanisms, they can also cause abnormal growth and increase the risk of breast cancer; parabens are also potentially working in relation to aryl hydrocarbon receptors [30,48,50,51,52].

**Figure 6:**
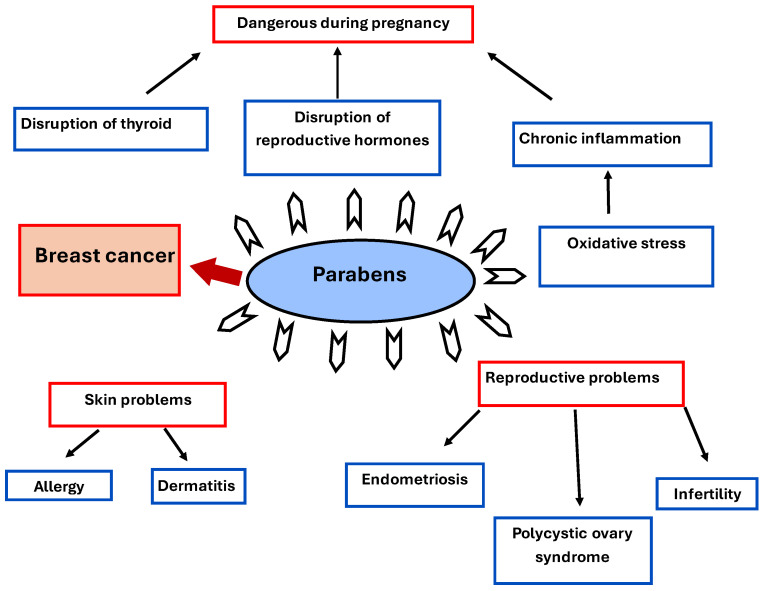
Parabens’ side effects on human health.

They have carcinogenic properties and show other side effects on human health besides breast cancer, as shown in Figure 6, “Parabens’ side effects on human health”. Reproductive problems include infertility, endometriosis, and polycystic ovary syndrome (PCOS). Skin problems are present in people with sensitive skin, and they are vulnerable to dermatitis and allergic reactions. Parabens are dangerous during pregnancy because of the disruption of thyroid and reproductive hormones [48].

### 3.3. Phenols

Phenols are found in cosmetics, shampoos, and plastics. Currently, thanks to the introduced restrictions, BPA cannot be used in personal hygiene products, but it can still seep into them from bottles. These compounds, apart from their endocrine-disrupting ability, are also suspected of having carcinogenic effects. Bisphenol A is the most explored among the group of phenols, classified as a reprotoxic substance, and causes reproductive diseases; however, the WHO does not classify it as carcinogenic. In relation to breast cancer, Bisphenol A (BPA), Bisphenol S and F (BPS, BPF), 4-Octylphenol (OP), 4-Nonylphenol (NP), and 2,2-bis(4-hydroxyphenyl)-1,1,1-trichloroethane (HPTE) have been reported. These compounds have a wide range of uses but are also subject to strict supervision, especially BPA. It cannot be found in baby bottles or in packaging intended for use by children under three years of age due to the ban on its use in such products. Regulation (EC) No. 1935/2004 specifies the permissible concentration of BPA in contact with food. Its migration limits in food are up to 0.05 mg/kg [15,51,53,54].

In addition to their estrogenic activity, there are also many reports about their possible effects on pregnant women. These substances can mimic hormonal functions by inhibiting receptors or related signaling pathways. Phenols cause a decrease in progesterone levels, which suggests that they are antagonistic to hormonal receptors. They are associated with progesterone mRNA expression via ERα signaling and interference with the transport of cholesterol to the mitochondria. They are suspected of disrupting steroidogenesis. All their hormonal disruption makes them antagonists of AhR, PRG, and AR and agonists of ERα, ERβ, and GPERs. Similarly to PCBs, in addition to their ability to bind to hormonal receptors, their mechanisms are correlated with HER-2, P53 Protein, Cytochrome P450, and oxidative stress [51,55,56,57].

BPS, a BPA-related compound, has become the focus of attention. It can induce the migration of cancer cells and is closely linked to acute cytotoxicity, neurotoxicity, immunotoxicity, and cardiovascular toxicity [56,57].

The results of the studies on the association between EDCs and breast cancer vary. The literature on BPA also shows varying results. Some studies show evidence against the association between BPA and breast cancer risk. On the other hand, animal studies show increased breast cancer risk when exposure to BPA begins in utero during pregnancy. One scientific report revealed that BPA exposure inhibited keratin 14 (KRT14) gene expression, thereby affecting MCF-7 cell proliferation, migration, and invasion. This suggests that KRT14 is an absolute determinant of bisphenol-induced breast cancer development. This gene, expressed in the tumor, represents a poor prognostic marker, particularly in invasive tumors [31,32,58,59].

Excessive stimulation of hormone-sensitive receptors by bisphenol A induces inflammation, leading to increased levels of ROS. Increased levels can damage DNA, triggering mutations in the p53 checkpoint enzyme and inducing the development of oncogenes. Approximately 50% of all cancers, including breast cancer, have a p53 mutation, which promotes tumorigenesis and metastasis. It is hypothesized that, in addition to the receptors mentioned above, BPA may also affect androgen receptors, thyroid hormone receptors, and other endocrine signaling pathways. However, more research is needed to confirm this hypothesis [8].

### 3.4. 2,3,7,8-Tetrachlorodibenzo-p-dioxin (TCDD)

2,3,7,8-Tetrachlorodibenzo-p-dioxin (TCDD) is a by-product of industrial processes, waste incineration, and fossil fuel combustion. It enters the environment by discharging into surface water and diffusing into the air. This substance belongs to the dioxin family and is known to be the most toxic, and it is classified as a carcinogen. Among the compounds presented, this poses the greatest threat. It can cause DNA damage, resulting in breast cancer, through estrogenic activity, AhR receptor activity, oxidative stress, regulation of Cytochrome P450 genes, and increased expression of p53 [34,60,61].

Commission Regulation (EU) 2023/915 of 25 April 2023 introduced the permissible concentrations of these compounds. An additional regulation determines different levels of action when the values approach the maximum permissible standards. Maximum levels in dairy products were designated at 2.5 pg/g of fat in meat, 1.25–5.0 pg/g of fat in oils, from 0.75 pg/g to 1.5 pg/g of fat, and 3.5–6.5 pg/g of fat in fish. Therefore, preventive measures have been introduced. TCDD has also been classified as a priority substance with a particularly high risk, and the standard for surface water is 0.0001 pg/L. To better protect the environment from production-related pollution, standards have been set for the chemical industry, waste incineration plants, metallurgy, and power plants, where the maximum permissible TCDD emission is 0.1 ng TEQ/m^3^ [34,62,63,64].

TCDD is an AhR agonist. This receptor, associated with the compound in question, causes tumor enlargement. Its ligands are also suspected to be inhibitors of breast cancer and act as anti-estrogens. Its relationship with estrogenic activity is related to the Aryl hydrocarbon receptor. Hormones can bind to estrogen-responsive elements (EREs) or in protein–protein interactions, such as AhR. In breast cancer, proteasome-dependent degradation of ERα is caused by the induction of TCDD, which promotes the formation of the AhR complex. This results in the displacement of ERα when TCDD is activated, as well as degradation of both ERα and AhR. The activity of cytochrome P450 is also influenced by dioxin, which affects estrogen metabolism [60,65,66,67].

**Figure 7:**
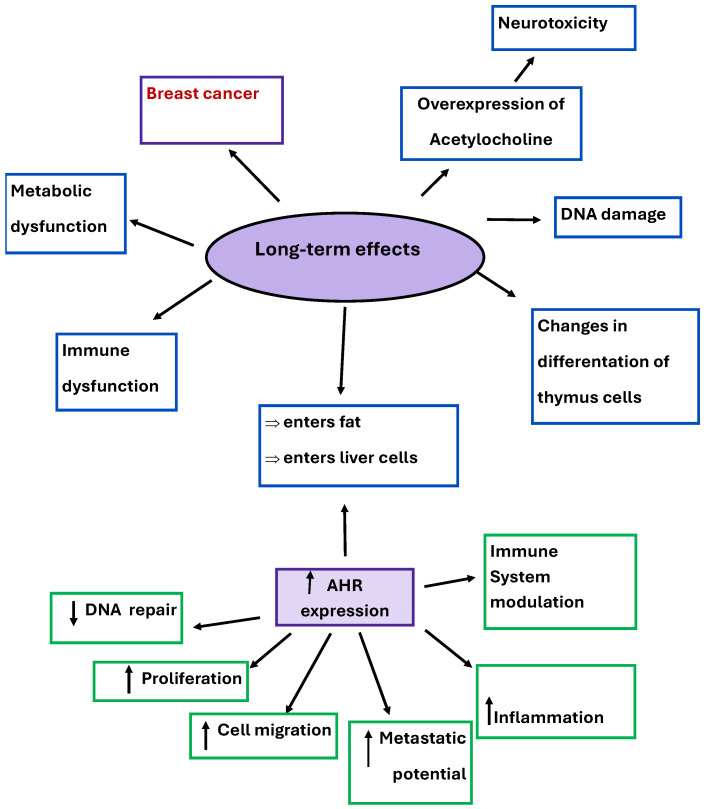
Long-term effects of TCDD and overexpression of AhR and its effects.

In addition to its role in tumorigenesis in breast cancer, it also has the ability to enter fat and liver cells through the AhR receptor, cause changes in thymus cell differentiation, and cause overexpression of acetylcholine, which results in neurotoxicity, metabolic and immune dysfunction, and DNA damage. Long-term effects of TCDD and the AHR’s relation to them are illustrated in Figure 7. The International Agency for Research on Cancer (IARC) classified TCDD as a class 1 carcinogen [60,61,68].

### 3.5. Diethylhexyl Phthalate (DEHP)

DEHP is widely used as a plasticizer, and its exposure can stimulate the proliferation of normal cells and drive PI3K, the AKT/mTOR signaling pathway, and cell cycle progression, especially after long-term exposure to this compound. It can bind to estrogen and progesterone receptors and mimic the functions of estrogen and progesterone, as was the case with other substances and their correlation with the estrogen receptor. PI3K/AKT signaling and continuous exposure to this substance are also associated with drug-resistant breast cancer [69,70,71,72].

DEHP is on the list of substances of very high concern (SVHCs, substances of very high concern). Since 2015, DEHP has been subject to authorization, which means that its use requires special consent. Since 2020, DEHP has been banned from use in consumer products in the EU, except for certain industrial applications. The use of DEHP in food packaging is limited to special applications, with a maximum migration of this compound into food of 1.5 mg/kg. There is also a ban on the use of DEHP in toys and children’s articles. It can constitute less than 0.1% of the weight of plastic. The use of DEHP in medical equipment is restricted [15,73,74,75].

As little as 10,000 nM of DEHP binds to progesterone receptors and causes an increase in the proliferation of cancer cells. The aryl hydrocarbon receptor mediates the proliferation of breast cancer cells through DEHP. A recent study has suggested that phthalates induce AhR/HDA6/cMyc transactivation in ER-negative breast cancers. Increased ROS content is related to AhR and also takes part in cancer development and proliferation [71,72,76].

Di(2-ethylhexyl) phthalate (DEHP) exposure is related to increased growth, proliferation, and angiogenesis in TNBC, as well as migration of breast cancer cells. Longer exposure also induces inflammation and proto-oncogene expression [71,72,77].

As a result of using DEHP in addition to breast cancer treatment, problems associated with pregnancy were identified. Mild issues, including weight gain and hormonal problems, were also encountered. Long-term exposure to EDCs causes problems in placental development. Exposure to this compound in children raises concerns about intellectual development, reduced intelligence, and attention deficit/hyperactivity disorders [71,78,79].

### 3.6. Impact of EDCs on Sensitive Populations

Endocrine-disrupting compounds disrupt hormonal balance and promote the development of cancer. Therefore, there is no doubt that individuals who are sensitive to toxic substances, such as infants and pregnant women, will be more susceptible to changes induced by environmental factors, which also include EDCs. Conducting a case–cohort study of EDCs in humans is quite difficult due to the variability in their concentrations over time. In 1976, a case of acute exposure to TCDD was reported in Italy. People living near an explosion that released 30 kg of the compound into the soil were exposed. Children were also among the exposed individuals. Twenty years later, there was a sudden increase in cancer rates, including breast cancer. A particularly high percentage of women, who were children at the time of the explosion, were affected. TCDD’s ability to bioaccumulate in fat cells led to an increased risk of breast cancer, which is known to be located at a higher percentage in adipose tissue [80,81].

Mammographic breast density, which measures the amount of fibroglandular tissue, is a useful diagnostic tool for assessing breast cancer risk. During puberty, adipose, epithelial, and stromal tissues in young girls’ breasts begin to develop. This is dependent on numerous hormonally driven processes. Exposure to an endocrine disruptor during this period can disrupt this process. Several studies have shown a positive correlation between EDCs and menarche and the risk of developing breast cancer. As suggested by one of the cohort studies of Chilean adolescents, childhood exposure to phthalates and phenols positively correlates with increased mammographic density during adolescence. The effect of EDC exposure on breast density has not been adequately studied, but there are scientific reports suggesting its relevance. This study is an example showing the potential impact of childhood EDC exposure on breast density in adolescent girls. A meta-analysis researching the association between mammographic density (MD) and breast cancer confirms the relevance of MD as a risk factor for breast cancer. It also states that the probability of a zero or negative association between MD and breast cancer is essentially zero (Daniela Bond-Smith et al., 2019 [82]). The samples for the cohort studies of Chilean adolescents were collected throughout puberty, which allowed the researchers to examine the associations of interest at more than one time point and identify potential windows of susceptibility (Lara S Yoon et al., 2022 [83]). The results suggest potentially varying windows of EDC susceptibility during puberty. This study also suggests that, although few cohort studies have evaluated the link between endocrine-disrupting chemicals and breast cancer composition in adolescents, the timing of breast development is related to breast composition. It also says that similar associations between EDCs and timing of breast development, as well as breast composition, should be expected (Lara S Yoon et al., 2022) [82,83,84].

BPA was detected in fetal plasma, placenta, and breast milk. Because the compound was used in the production of infant feeding bottles and was present in care products such as shampoos and cosmetics, the sources of microdose exposure were cumulative. It is known that, besides breast cancer, endocrine-disrupting chemicals cause several diseases. Both bisphenol A and bisphenol S have the ability to cross the placental barrier. By affecting the mammary gland, EDCs also directly affect breastfeeding in pregnant women who are exposed to these compounds. In pregnant rats, substances interacting with estrogen caused long-term changes in mammary gland morphology. A similar phenomenon could likely be observed in women, but for ethical and moral reasons, such studies cannot be conducted. We can only rely on measuring the levels of these compounds in the blood and urine, along with simultaneous imaging studies [85,86,87,88].

### 3.7. Simultaneous Exposure

A single exposure to a chemical compound is rarely observed. Living organisms are more often exposed to a mixture of chemicals; thus, the accumulation of small doses can lead to various health effects. Breast cancer caused by environmental exposure is a growing concern. The chemicals discussed in this article, particularly TCDD, parabens, and PCBs, have a strong environmental impact. Simultaneous exposure to multiple endocrine-disrupting chemicals leads to additive, synergetic, and antagonistic effects. This is especially shown in the disturbance of estrogen hormonal pathways. Estrogen receptors show additive and synergistic effects when exposed to a mixture of EDCs. Simultaneous exposure to phenols and parabens can lead to abnormal breast development and obesity, which are predisposing factors for breast cancer. Furthermore, continuous exposure to environmental toxins increases the risk of breast cancer by up to 70% to 95%. About half of cancers in women, including BC, are related to estrogen and androgen hormones [89,90,91,92,93].

Table 1 shows cumulative information from the entire article and its references on the effects of hormone receptor activation and mechanisms, in relation to EDCs. Columns represent the classification, receptor interaction, mechanisms or effects, and regulatory status of the discussed compounds. TCDD and PCBs were confirmed to be carcinogenic by the International Agency for Research on Cancer (IARC). Phenols, including Bisphenol A, are classified as reprotoxic substances, and parabens, recognized as hazardous, pose a significant health risk. PCBs and DEHP are potential threats to the environment and human health. Endocrine-active compounds have an affiliation with the estrogen receptor, which has also been proven in PCBs, TCDD, parabens, phenols, and DEHP. They are also AhR agonists (PCBs, TCDD, DEHP) or antagonists (parabens, phenols). Antagonism to progesterone receptors was exhibited in phenols and DEHP, while parabens are suspected to be antagonists. Phenols, parabens, and PCBs inhibit the androgen receptor [39,94].

Orally administered dioxins and PCBs were examined in studies looking at the effects of a mixture of endocrine-disrupting compounds on breast cancer. Epidemiological studies suggested an increased risk of breast cancer, or no risk, following acute exposure to these compounds, and this finding was confirmed. As can be easily seen in Table 1, the compounds discussed here act through similar hormonal regulatory mechanisms. They also utilize the same receptors, so they should be studied with consideration of their accumulation potential and the potential interactions that may occur in the human body if all of these compounds are present. Even if only a few compounds are detected, it is possible that they will act additively. In the case of antagonism and agonism at a single receptor, this effect may be offset. This relation still needs to be studied, but it shows promising research prospects [89].

## 4. Conclusions

Endocrine-disrupting chemicals, which are commonly used in the environment, pose a serious threat to human health, not only in connection with various types of cancer, but also in the proper functioning of the body by affecting the reproductive system and metabolism and through toxic effects on the human body.

All compounds included in this study were used in products that consumers deal with every day. There is a significant link between endocrine-disrupting chemicals and breast cancer. A significant number of EDCs are suspected to cause breast cancer, some of which are carcinogens. These compounds are receiving increasing attention, not only because of their possible carcinogenic effects but also because of their long-term effects.

Among parabens, polychlorinated biphenyls, diethylhexyl phthalate, and Phenols, 2,3,7,8-Tetrachlorodibenzo-p-dioxin poses the greatest threat to human health. It is important to expand our knowledge and the scope of research on other compounds, especially parabens, due to their potentially antagonistic progesterone, androgenic, and AhR receptor effects.

The cumulative effects of PCBs, parabens, TCDD, DEHP, and phenols were examined. No articles reporting combined studies were found. Instead, studies used single exposures to these substances or other compounds from this group. Research in this area is still far from being ideal, especially since these compounds are being studied not only in relation to breast cancer but also in relation to other types of malignancies.

The ability of these compounds to cause carcinogenic effects in breast tissue or to cause further cancer development by improving the proliferation and migration of cancer cells has been demonstrated. EDCs affect healthy tissues in different ways by binding to different receptors. More attention should be paid to these compounds to study their broader effects on the human body. Table 2 “Important information,” summarizes important issues presented in the article.

## Figures and Tables

**Table 1:** Receptors of endocrine-disrupting chemicals and effects of their activation.

Substance	Receptor Interaction	Mechanism/Effects	Regulatory Status	References
PCBs	- Agonists to ERα, ERβ, and GPER - AhR agonists -> inhibition of ER - Antagonists to AR	- Estrogen Receptor - HER-2 - Ki67 - P53 Protein - Progesterone Receptor - Aryl Hydrocarbon Receptor - Cytochrome P450 - Oxidactive Stress	- Resistant to biodegradation, - High bioaccumulation and biomagnification potential - Type 1 carcinogen	[30,36,38,39,40,66]
Parabens	- Potential antagonists to AhR - Potential antagonists to AR - Potential antagonists to PRG - Weak ERα agonists - Weak ERβ agonists, but stronger than ERα	- Estrogen-Related - Androgen-Related - Progesterone-Related - AhR-Related	- They are hazardous - Not classified as a carcinogen	[30,44,48,50,51]
Phenols	- Antagonists of AhR - Antagonists to PRG receptor - Antagonists to AR - Agonists to ERα, ERβ, and GPER	- Estrogen Receptor - HER-2 - P53 Protein - Progesterone Receptor - Aryl Hydrocarbon Receptor - Cytochrome P450 - Oxidactive Stress	- BPA is classified as a reprotoxic substance - Not classified as a carcinogen	[30,51,55,56]
TCDD	- Strong agonist to AhR - Antagonist to ERα	- Estrogen Receptor - Cytochrome P450 - Aryl Hydrocarbon Receptor - P53 Protein	- Classified as type 1 carcinogen	[40,60,61,65,66,67]
DEHP	- Agonist to ERα - Agonist to AhR - Antagonist to PRG	- Estrogen Receptor - Progesterone Receptor - Aryl Hydrocarbon Receptor	- Substance of very high concern (SVHC)	[70,71]

**Table 2:** Important information.

Important Information
Endocrine-disrupting chemicals: - Are a heterogeneous group of substances, such as polychlorinated biphenyls, parabens, phenols, 2,3,7,8-tetrachlorodibenzo-p-dioxin, diethylhexyl phthalate, and bisphenol A. - TCDD, parabens, and PCBs have strong environmental impacts. - Can act as agonists or antagonists to hormonal receptors. - Are present in various industries. - Can bioaccumulate and disturb hormonal balance. - Can act as carcinogens.
- Maximum allowable doses are regulated by the European Union: REACH, CLP. - The IARC classifies carcinogenic compounds and divides them into categories. - The relationship between dose and effect is not necessarily linear, and each compound in this group may be metabolized differently; thus, the minimum effective concentrations of these compounds should be approached with caution.
Breast cancer subtypes are characterized by the presence of hormonal receptors, which are ER, PR, HER2, and Ki67 receptors. Subtypes of breast cancer are presented in Figure 2.
- Each of the analyzed compounds has been suspected of causing breast cancer, as well as other harmful long-term effects (Table 1, Figure 5, Figure 6 and Figure 7).
- EDCs, which are also environmental pollutants, are extremely dangerous to sensitive populations such as pregnant women, infants, and children.
- Simultaneous exposure to chemicals is observed more often than single exposure, but research in this area is still lacking. - It leads to additive, synergetic, and antagonistic effects.

## Data Availability

The authors confirm that the data supporting the findings of this study are available within the article and from the corresponding author [L.C.] upon reasonable request.
